# Patterning Planar, Flexible Li-S Battery Full Cells on Laser-Induced Graphene Traces

**DOI:** 10.3390/nano15010035

**Published:** 2024-12-29

**Authors:** Irene Lau, Adam I. O. Campbell, Debasis Ghosh, Michael A. Pope

**Affiliations:** 1 Quantum Nano Centre, Department of Chemical Engineering, University of Waterloo, Waterloo, ON N2L 3G1, Canada; 2 Centre for Nano & Material Sciences, Jain (Deemed to be University), Jain Global Campus, Bangalore 562112, India; g.debasis@jainuniversity.ac.in

**Keywords:** laser-induced graphene, Li-electroplating, Li-S batteries, planer-interdigitated electrodes, flexible batteries

## Abstract

Laser conversion of commercial polymers to laser-induced graphene (LIG) using inexpensive and accessible CO_2_ lasers has enabled the rapid prototyping of promising electronic and electrochemical devices. Frequently used to pattern interdigitated supercapacitors, few approaches have been developed to pattern batteries—in particular, full cells. Herein, we report an LIG-based approach to a planar, interdigitated Li-S battery. We show that sulfur can be deposited by selective nucleation and growth on the LIG cathode fingers in a supersaturated sulfur solution. Melt imbibition then leads to loadings as high as 3.9 mg/cm^2^ and 75 wt% sulfur. Lithium metal anodes are electrodeposited onto the LIG anode fingers by a silver-seeded, pulse-reverse-pulse method that enables loadings up to 10.5 mAh/cm^2^ to be deposited without short-circuiting the interdigitated structure. The resulting binder/separator-free flexible battery achieves a capacity of over 1 mAh/cm^2^ and an energy density of 200 mWh/cm^3^. Unfortunately, due to the use of near stoichiometric lithium, the cycle-life is sensitive to lithium degradation. While future work will be necessary to make this a practical, flexible battery, the interdigitated structure is well-suited to future operando and ex situ studies of Li-S and related battery chemistries.

## 1. Introduction

In 2014, Lin et al. reported the accidental discovery that exposing commercial polymers, such as Kapton tape, to a CO_2_ laser led to the formation of a porous graphene-like foam widely referred to as laser-induced graphene (LIG) [1]. The simplicity of the approach and the easy access to these inexpensive lasers, widely used for cutting and milling in machine shops across most research institutions, have led to an explosion in research in this area related to different materials that can be laser converted and devices that can be rapidly prototyped [2,3,4,5,6,7].

In the simplest case, the high conductivity of the LIG alone can lead to laser-written, flexible traces for circuits such as RFID tags, antennas, and resistive heaters [8,9,10,11], or its hydrophobic nature can be exploited to create superhydrophobic surfaces via patterning [12,13]. The high specific surface area of the porous LIG structure formed make it particularly interesting for electrochemical applications. For example, flexible, interdigitated electrochemical double-layer capacitors (EDLCs) have been demonstrated with capacitances ranging from 1 to 100 mF/cm^2^ [1,14,15,16,17]. By coating redox active materials, such as PANI, MnO_2_, and FeOOH [18], or Co_3_O_4_ [19] onto/within the porous LIG fingers by electrodeposition, the capacitance has been boosted by another order of magnitude. Subsequent work showed that electrocatalysts could be embedded in the fingers by either embedding catalysts in the polymer precursor and lasing to simultaneously generate the catalyst-decorated LIG structure [20,21] or casting catalyst precursors into the LIG pores and lasing them again to in situ form the catalyst. The resulting electrodes have been used for electrolyzers and as air cathodes for zinc-air batteries [22]. Recently, Ghosh et al. [23,24] demonstrated flexible aqueous zinc-ion batteries based on electrodeposited zinc anodes and metal oxide cathodes with patterned LIG as the current collector.

Engineered LIG has recently shown significant promise in enabling dendrite-free lithium (Li) and sodium (Na) deposition, making it a viable candidate for Li/Na-ion batteries. For instance, Yi et al. [25] developed a 3D hierarchical structure comprising a copper substrate, an array of flexible polyimide (PI) pillars, and porous LIG formed on the walls of the polymer pillars. This structure was created by directly scribing a PI film on a copper support using multiple laser scans. The resulting material exhibited a reduced Li nucleation overpotential compared to bare copper, attributed to the high density of defects and the presence of heteroatoms in the LIG. Additionally, Xiao et al. [26] enhanced the performance of LIG on copper substrates by incorporating lithiophilic MnOx nanoparticles, achieving dendrite-free Li deposition with an impressive rate capability of up to 40 mA/cm^2^. Similarly, the Tour group demonstrated that the silicon-based adhesive of PI tape could be transformed into a porous SiOx film with a small amount of LIG directly deposited onto a copper current collector, which was successfully utilized in an anode-free lithium metal battery [27]. Recently, Xiao et al. [28] also explored sodium-ion batteries by modifying LIG on copper supports with Sn nanoparticles, achieving a reduced Na nucleation overpotential. However, while these configurations employing copper current collectors and additive-free systems show considerable promise, they lack design flexibility, which could limit their adaptability for applications in flexible devices.

In this work, we demonstrate a simple approach to create interdigitated lithium sulfur (Li-S) batteries using LIG patterned on a commercial PI substrate. The Li-S battery chemistry promises a significant improvement in energy density over Li-ion batteries due to the high theoretical capacity of both lithium (3860 mAh/g) and sulfur (1672 mAh/g) as active materials while achieving a moderate cell voltage of ~2.15 V. These attributes, combined with the low cost and abundance of sulfur, make the Li-S battery a promising alternative to Li-ion [29]. However, there are several challenges that have hindered commercialization [30,31,32,33]. Critical issues include the electrically insulating nature of sulfur and the discharge products Li_2_S_2_/Li_2_S within the cathode, and the dissolution of the intermediate Li polysulfide species (LiPSS), which can shuttle to/from the anode. This results in rapid capacity fade and self-discharge. Furthermore, the highly reactive Li metal anode forms porous and dendritic structures during cycling, resulting in the need for a significant excess of Li and electrolyte to sustain the formation of the ever-evolving solid electrolyte interface (SEI) [34,35].

Over the last decade, significant progress has been made to mitigate these challenges, moving Li-S batteries closer to commercialization. For example, strategies to couple high surface area supports with various polysulfide scavengers or selective membranes now lead to stable, high-loading cathodes (>3–4 mg/cm^2^) that can achieve >1000 mAh/g for nearly 1000 cycles [36]. While there are still significant challenges to overcome—especially on the anode side—a more recent flurry of investigations focused on the development of Li protection strategies [34,37,38] suggests that stable, high-energy-density full cells might be on the horizon through the use of improved electrolyte additives [39,40,41,42], host structures [39,43,44,45,46], and artificial SEIs [39,47,48,49,50,51].

Current research is now moving towards addressing the remaining challenges at the full cell level. Ensuring the commercial viability of Li-S batteries necessitates minimizing the excess Li with improved plating striping efficiency in a full cell. However, comprehensive testing of full Li-S cells with nearly stoichiometric lithium to evaluate performance and anticipate potential failure modes in practical battery scenarios is rare. While alternative approaches for developing anode-free and solid-state Li-S batteries [52,53] have demonstrated promising initial outcomes, they are still in their early stages of laboratory research [54,55]. Additionally, limitations in available materials, laboratory equipment, and the coin/pouch cell geometry hinder testing with stoichiometric lithium and lean electrolyte, as well as in situ analysis.

Given these challenges and the promising potential of Li-S batteries for future flexible and miniaturized applications, we present a proof-of-concept flexible Li-S battery. This design employs polyimide-derived interdigital patterned LIG as both the current collector and a porous conducting matrix for sulfur dispersion, as well as a substrate for lithium electroplating, using a rapid, scalable, and cost-effective prototyping method. The pseudo 2D, interdigitated nature of the cell provides the ability to monitor operation by in situ microscopy or spectroscopy while providing a platform for future flexible and miniaturized battery systems. Herein, we outline our initial system design with a high sulfur content and near stoichiometric lithium, with a high initial capacity, laying a foundation to further improve the cycle stability with suitable structural modification such as implementing polysulfide retention or Li-protection strategies for practical full cell application. The interdigitated cell design also offers flexibility for customized form factors and an optically and spectroscopically accessible platform for future operando and ex situ studies of factors such as sulfur speciation and lithium metal plating morphology.

## 2. Materials and Methods

### 2.1. Electrode Patterning

**Figure 1:**
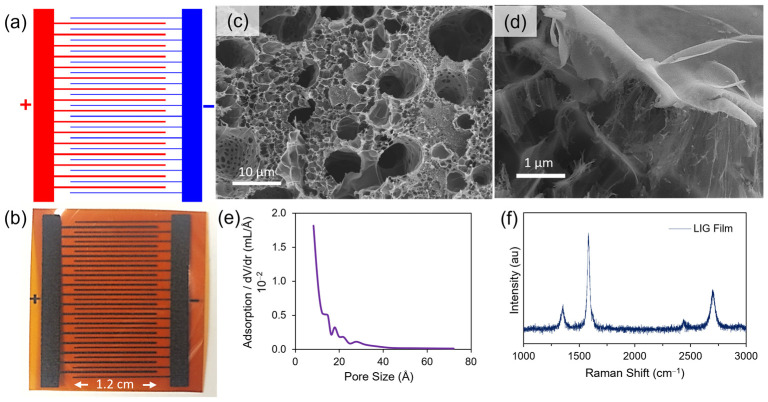
(**a**) Laser-scribing pattern of device on computer software for laser and (**b**) completed device after scribing. There are 17 anode fingers interleaved with 16 cathode fingers that are approximately 175 µm (prior to Li plating) and 315 µm in width, respectively. The distance between each finger of opposite polarity is 310 µm (prior to Li plating). The overlap distance between anode and cathode fingers is 1.2 cm, leading to a geometric area of 0.357 cm^2^ and 0.605 cm^2^, respectively. The design was explicitly chosen such that the anode fingers would be thinner in order that more Li metal can be plated onto the LIG without short circuiting the final device. (**c**,**d**) SEM micrographs depicting hierarchical pore structure of LIG at different magnifications. (**e**) Pore size distribution of LIG material obtained by ethanol vapor adsorption analyzer. (**f**) Raman spectrum for LIG on PI with two laser passes at 10% power and 30 mm/s speed.

The method of creating LIG on polyimide as published by the Tour group^8^ was adopted in the preparation of the electrodes for the flexible battery. Kapton© (HN polyimide) film with a thickness of 127 µm was purchased from McMaster-Carr to be used as the substrate and source of carbonizable material for the electrodes. The polyimide (PI) film was used as is without modification, and a CO_2_ laser system (Boss Laser LS-1416, 50 W, 10.6 µm) was used for the laser-patterning of the electrodes. The interdigitated pattern of the electrodes can be seen in Figure 1a, where the red area corresponds to the cathode of the battery and the blue area corresponds to the anode of the battery. The two sets of electrodes are scribed separately, with the cathode first and then the anode after sulfur deposition. For both sets of electrodes, laser induction occurred in raster scanning mode with an x-swing at a scan speed of 30 mm/s and approximately 10% power (5 W). Due to uncertainties associated with daily laser alignment and power fluctuations, the exact power chosen was determined by scribing a test substrate at various powders near 10% and choosing the power that yielded the maximum conductivity. The final laser-scribed device is seen in Figure 1b.

### 2.2. Cathode: Sulfur Deposition onto LIG

Sulfur crystals were heterogeneously nucleated onto the surface of the LIG electrodes through a reduction in the solubility of sulfur by the cooling of a saturated solution of sulfur (purum p.a., ≥99.5%, from Sigma Aldrich, Oakville, ON, Canada) in dimethyl sulfoxide (DMSO, ≥99.7%, Fisher Scientific, Toronto, ON, Canada) with a concentration of 40 mg/mL. This solution was first heated up to 180 °C on a hot plate in order to dissolve the sulfur in the DMSO under magnetic stirring. The cathode-scribed sample was placed with the electrodes facing down in a glass petri dish and weighed down by a glass plate in the dish, which were all preheated to 130 °C on a hot plate. The heat was shut off on the hot plate after 750 µL of the hot S/DMSO solution was injected between the sample and the petri dish. The petri dish, sample, and solution were allowed to cool on the hot plate for 15 min. The sample was then gently but thoroughly rinsed with deionized (DI) water to remove any excess solution and then dried at 110 °C for 15 min in air on the hot plate. After drying, the heterogeneously nucleated sulfur crystals were melted through a brief flash heating of ~15 s on the hot plate at 165 °C, where, upon contact with the hotplate, the sulfur crystals on the sample would imbibe into the LIG pore structure. This process was repeated for up to four depositions to achieve loadings around 4 mg/cm^2^ as discussed later.

### 2.3. Anode: Lithium Electrodeposition onto LIG

After the deposition of sulfur into the set of electrode fingers for the cathode, another set of LIG electrode fingers was patterned for the anode. For the electrodeposition of lithium, the sample was placed in an argon-filled glove box, and the setup as described in Figure S1 was used. Lithium ribbon (length × width = 5 × 4 cm, thickness of ~0.75 mm, Sigma Aldrich, Oakville, ON, Canada) was used as the counter electrode (CE), and the working electrode (WE) was the LIG anode fingers themselves. Brass contacts (Ultra-Formable 260 Brass Sheet, McMaster-Carr, Chicago, IL, USA) were used for the contact of both the WE and CE. The plating electrolyte used was 1 M lithium perchlorate (LiClO_4_, 99+%, Acros Organics, Fisher Scientific, Toronto, ON, Canada) in 1:1 (*v*/*v*) ethylene carbonate (EC, anhydrous 99%, Sigma Aldrich, Oakville, ON, Canada)/dimethyl carbonate (DMC, anhydrous ≥ 99%, Sigma Aldrich, Oakville, ON, Canada), which was used as is.

To aid the plating of lithium, a reverse pulse technique (RPP) and Ag NPs were used as specified. The specifics of the RPP parameters are detailed in Table S1, and a plating capacity of 10.5 mAh/cm^2^, which is equivalent to a 2× stoichiometric amount of lithium relative to the loading of approximately 4 mg/cm^2^ of sulfur, was used for the LSFB. Colloidal Ag NPs were synthesized by the reduction of AgNO_3_ (ACS, 99.9+%, metals basis, Alfa Aesar, Haverhill, MA, USA) by citric acid (trisodium salt dihydrate, 99% pure, Acros Organics, Fisher Scientific, Toronto, ON, Canada), according to Ratyakshi [56]. To imbibe the Ag NPs into the LIG electrodes, the entire laser-scribed sample was submerged in a bath of ethanol for 10 min in order to wet and displace air from the porous LIG network. Afterwards, the sample was submerged into the colloidal bath of Ag NPs for 10 min, which solvent exchanges the Ag NP dispersion with ethanol. The sample was then heated on a hot plate at 110 °C for 15 min to remove all water from the sample prior to being placed in the Ar-filled glove box for Li electrodeposition.

### 2.4. LIG-Based Full Cell

After Li electrodeposition, the samples were rinsed with 1:1 (*v*/*v*) EC/DMC without the LiClO_4_ salt to remove all traces of excess salt. Then, the samples were fully dried by vacuum in order to remove excess solvent. Once dried of the carbonate-based solvent, the samples were cycled using the cycling setup illustrated in Figure S5a in a room temperature ionic liquid (RTIL)-based electrolyte composed of 1 M lithium bis (trifluoromethylsulfonylimide) (LiTFSI, 99.95%, Sigma Aldrich, Oakville, ON, Canada) in 1-ethyl-3-methylimidazolium bis (trifluoromethylsulfonylimide) (EMImTFSI, 99%, IoLiTec, Heilbronn, Germany).

### 2.5. Li vs. Li Symmetric LIG-Based Cell

The preparation of a LIG-based symmetric Li vs. Li cell involved the laser scribing of both sets of LIG electrodes in the interdigitated form, followed by the Ag NP imbibition. RPP was used for the plating of Li on both sets of electrodes to a capacity of 5.25 mAh/cm^2^ (equivalent to 1× stoichiometric Li relative to the sulfur loading of approximately 4 mg/cm^2^) in the same plating setup in Figure S1. After Li plating on both sets of electrodes, the sample was rinsed with 1:1 (*v*/*v*) EC/DMC to remove excess salt prior to cycling in the RTIL-based electrolyte, 1 M LiTFSI in EMImTFSI. The symmetric cell was cycled in the setup depicted in Figure S5a.

### 2.6. S/LIG vs. Li Full Cell

On a larger area of LIG film, sulfur was deposited using the described deposition method with four deposition cycles to a loading of 4 mg/cm^2^ and a final weight percentage of ~70%. The S/LIG material was then scraped off of the PI substrate and then pressed into an electrode (1/2” diameter) against an Al foil current collector with a very low pressure, only sufficient enough to form a very thin pellet with an areal loading of 4 mg/cm^2^. The cathode was then transferred into an Ar-filled glovebox and used to make a CR2032 coin cell. The coin cell components and architecture are as shown in Figure S5b with a glass fiber membrane (pore size of 2.7 µm, thickness of 160 µm, Sigma Aldrich), Li foil, and 100 µL of electrolyte. Two electrolytes were used for comparison, the RTIL-based electrolyte used for the LIG-based full cell (1 M LiTFSI in EMImTFSI) and an ether-based electrolyte, 1 M LiTFSI in 1:1 (*v*/*v*) 1,3-dioxolane (DOL, anhydrous 99.8% with ~75 ppm BHT as inhibitor, Sigma Aldrich, Oakville, ON, Canada)/1,2-dimethoxyethane (DME, anhydrous 99.5%, Sigma Aldrich). After assembly, the cells were taken out of the glovebox for testing.

### 2.7. Material Characterization

The pore structure of the LIG material was characterized through scanning electron microscopy (SEM, FEI Quanta FEG 250 ESEM with energy dispersive X-ray spectroscopy (EDX)) at an acceleration voltage of 20 kV, and surface area measurements were conducted using a DVS Advantage Surface Measurement System, between partial pressures of P/Po = 0–0.5 using ethanol as the adsorbate. Raman spectroscopy was conducted using a Horiba Jobin-Yvon HR800 Raman system equipped with an Olympus BX 41 microscope with a 532 nm laser operating at 50% laser power (50 mW total power). The determination of sulfur and Ag NP wt% was determined through thermal gravimetric analysis (TGA, TA Instruments Q500, New Castle, DE, USA). TGA was carried out for the S/LIG and Ag NP/LIG material from 25–800 °C at a ramp rate of 10 °C/min, and the samples were held at 100 °C for 30 min prior to further temperature increase in order to remove excess water. S/LIG samples were tested under nitrogen gas, while Ag NP/LIG samples were tested in air. The calculations for the weight fractions are determined as described in the Supporting Information.

To observe the sulfur distribution within the pores of the LIG fingers, the sulfur cathode fingers (on PI) were encased in poly(methyl methacrylate) (PMMA, MW = 35,000, Acros Organics, Fisher Scientific, Toronto, ON, Canada) after four sulfur deposition cycles. The encapsulation was formed through the dissolution of PMMA in acetone (~5 mg/mL), followed by the drop-casting of the PMMA solution onto the LIG fingers. After the cast solution was dried, the sample was characterized through EDS.

### 2.8. Electrochemical Characterization

For the electrodeposition of Li, the constant current, alternate pulse galvano cycling programs, and electrochemical impedance spectroscopy (EIS), the EC-Lab software version 11.33 of a BioLogic SP-300 instrument was used. EIC was conducted between 5 MHz and 100 mHz in potentiostatic mode. For the cycling of the LSFB full cell, Li vs. Li symmetric cell, and S/LIG vs. Li coin cells, the cells were connected to a battery tester BTS 3000, Neware. The full cells were cycled by galvanostatic cycling at 0.1 C between 3 V and 1.5 V, while the symmetric cells were cycled between +1.5 V and −1.5 V, at the same current as the full cells for a consistent current density. While most symmetric Li cells in a coin cell format would be cycled between +1 V and −1 V at most, the wider voltage range chosen in this instance is to accommodate any ohmic drop due to the distance between the sets of LIG fingers.

## 3. Results and Discussion

### 3.1. LIG Electrode Properties

Figure 1a–b illustrates the LIG interdigitated pattern written into commercial PI tape prior to deposition of sulfur and lithium metal. There are 16 cathode fingers sandwiched between 17 anode fingers. The width of each cathode finger is 315 ± 50 μm, and each anode finger is 175 ± 40 μm with a distance between each adjacent finger of opposite polarity being 300 ± 50 μm (prior to Li plating). Examples of the widths measured by SEM are shown in Figure S1. The sets of fingers have an overlapping distance of 1.2 cm. The anode fingers were thinner such that more Li metal could be introduced onto the LIG without short-circuiting the final device. As illustrated by the SEM images and pore-size distribution shown in Figure 1c,d, the LIG electrodes are hierarchically porous as a result of the rapid gas evolution caused by photothermal conversion of PI to LIG upon exposure to the 10.8 μm wavelength laser beam. Many macropores as large as 10 μm can be observed (Figure 1c), with the pore walls being both sheet-like in morphology with some areas containing thin fibrous structures 10–100 nm in diameter. Ethanol vapor adsorption revealed a distribution of micro- and mesopores (Figure 1e) with a mean pore size of 1.28 nm (in the range of pores below 8 nm), which led to a specific surface area of about 183 m^2^/g (see Figure S3 for the adsorption curves and BET transform). Raman spectroscopy (Figure 1f) confirmed the characteristic D, G, and 2D peaks for graphitized carbon observed at 1349.39 cm^−1^, 1583.02 cm^−1^, and 2699.80 cm^−1^, respectively. The spectrum is consistent with the results of similar LIG work and confirms the graphitic structure, which resembles that of multi-layer graphene [1,18,57,58].

### 3.2. Sulfur Deposition Approach onto Cathode Fingers

**Figure 2:**
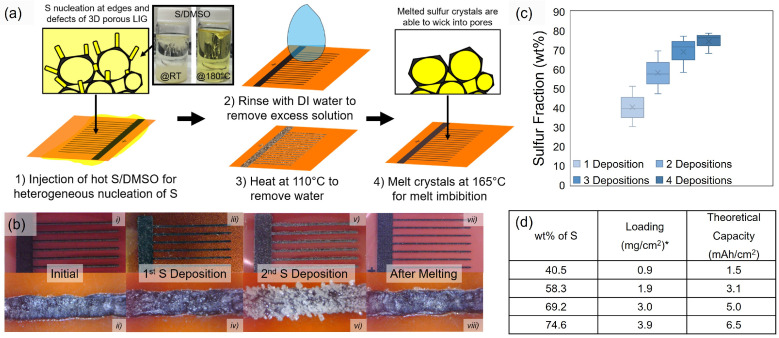
(**a**) Schematic of the two-step sulfur deposition process involving the heterogeneous nucleation of sulfur onto the LIG followed by melt imbibition, which can be repeated until a desired loading. The inset image of the two S/DMSO solutions depicts sulfur’s varying solubility within DMSO at room and elevated temperatures. (**b**) Digital images of LIG electrodes where (**i**,**ii**) show pristine LIG electrodes prior to any sulfur deposition and (**iii**,**iv**) depict the electrodes after the first deposition of sulfur and the unmelted crystals. (**v**,**vi**) show the electrodes after the second deposition of sulfur, while (**vii**,**viii**) depict the LIG electrodes after the sulfur crystals are melted. Subsequent depositions appeared similarly. (**c**) Box and whisker plot for the TGA results of sulfur wt% as a function of the number of depositions. (**d**) Average wt% of sulfur and the corresponding loadings and theoretical capacities obtainable as per (**c**). * Loading calculated is dependent on the area of the sulfur cathode alone in accordance with Equation (S1).

As shown in Figure 2a, in order to selectively introduce sulfur into the porous LIG cathode fingers, we first only scribed the cathode side and hypothesized that the well-known [43,59,60], good wetting properties of sulfur on carbon might facilitate a selective deposition process via heterogeneous nucleation and growth [59,60,61]. As shown in Figure 2a, hot DMSO has a high capacity for dissolving elemental sulfur. A solution concentration as high as 40 mg/mL sulfur in dimethyl sulfoxide (DMSO) is achievable at 180 °C, forming a translucent yellow solution. On the other hand, at room temperature, DMSO has a much lower solubility—especially compared to commonly used solvents such as tetrahydrofuran. Upon cooling the hot S/DMSO, in the absence of the LIG substrate, sulfur precipitates homogeneously from solution, forming large crystals that sediment rapidly to the bottom of the vial. As shown in Figure 2b(iii,iv), when the patterned PI sheet is exposed to a hot S/DMSO bath, during cooling, we observe the formation of small crystallites that heterogeneously nucleate mainly on the LIG pattern with only a few crystals being found on the PI itself. We attribute this to the preferential wetting properties of sulfur on carbon versus sulfur on the PI. It is well known that sulfur wets carbon [59]. The few sulfur crystals on the PI can be easily rinsed off with water, while those on the porous LIG electrodes are more adherent—as they are likely embedded partially in the porous network.

After the sulfur-decorated fingers were briefly heated to 180 °C, as shown in Figure 2b(vii,viii), the crystals disappeared. Thermal gravimetric analysis (TGA) indicated that the sulfur had not evaporated/sublimed but was still present at a fraction of ~40 wt% sulfur (Figure 2c,d). When this process was repeated, as shown in Figure 2b, the density and size of the sulfur crystals present on the outside of the fingers, as evidenced by optical microscopy, increased from the first to the second cycle. Homogenous growth on a pre-existing sulfur solid is more favorable than the heterogeneous nucleation of sulfur on the bare LIG. This second deposition and melting cycle led to 58% sulfur within the LIG. With four deposition cycles, 75 wt% sulfur can be achieved, which exceeds the sulfur fraction in most reports of Li-S batteries. The loading ranges (approximately 0.9 to 3.9 mg/cm^2^) in Figure 2d correspond to the theoretical capacities of 1.5 to 6.5 mAh/cm^2^, which is the range of current large-format Li-ion batteries. Furthermore, for large-format sulfur cells, moderate loadings of 3–5 mg/cm^2^ are expected to yield practical energy densities of 400–500 Wh/kg, while higher loading typically leads to no significant enhancement of the energy density due to poorer active material utilization and/or it typically requiring the use of heavier 3D current collectors [62]. As such, the target loading for the LIG battery system was 3–5 mg/cm^2^. This novel patterning approach of selective heterogeneous nucleation and melt imbibition of sulfur into the porous LIG builds on the traditional method of sulfur melt infiltration developed by Nazar et al. [43] for bulk powder-based electrode materials.

**Figure 3:**
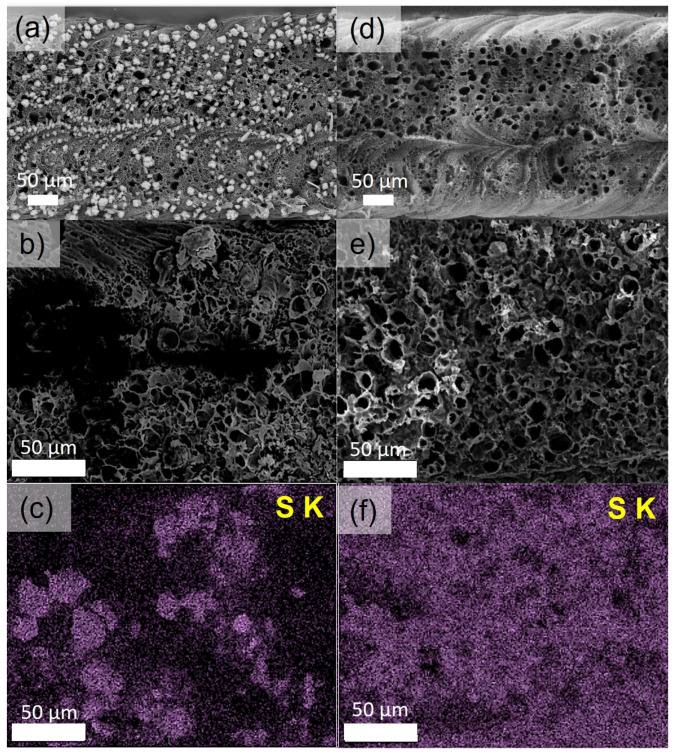
SEM/EDS mapping of sulfur cathodes before and after melt imbibition: (**a**,**b**) Backscattered electron contrast images at different magnifications to show contrast between sulfur and carbon. In (**a**), the brighter contrast corresponds to sulfur, which has a higher molecular weight and more intense backscattering compared to carbon. In (**b**), this effect is blurred by charging of the sulfur at higher magnifications. (**c**) Corresponding EDS map to (**b**). (**d**,**e**) Backscattered electron contrast of sulfur cathodes after melt imbibition illustrating the uniform distribution of sulfur. (**f**) Corresponding EDS map to (**f**). Scale bars in all images = 50 µm.

As shown in Figure 3, the distribution of sulfur within the LIG was determined by scanning electron microscopy (SEM) and energy dispersive X-ray spectroscopy (EDS). Prior to melting, large crystals of sulfur are found (~10–25 µm diameter) on the surface of the LIG (Figure 3a). These crystals shrink rapidly under the high vacuum conditions of the SEM, and thus, they are smaller than the crystals observed by optical microscopy. After melt imbibition, no large crystals are observed, as shown in Figure 3d. The EDS mapping illustrates the presence of sulfur crystals (Figure 3c) that melt to uniformly wet the surface of the LIG (Figure 3f) after the met infiltration. At 165 °C, the temperature at which the samples were heated and at which molten sulfur is the least viscous, molten sulfur is drawn into the carbonaceous pores by capillary action [59].

### 3.3. Lithium Electrodeposition onto LIG

**Figure 4:**
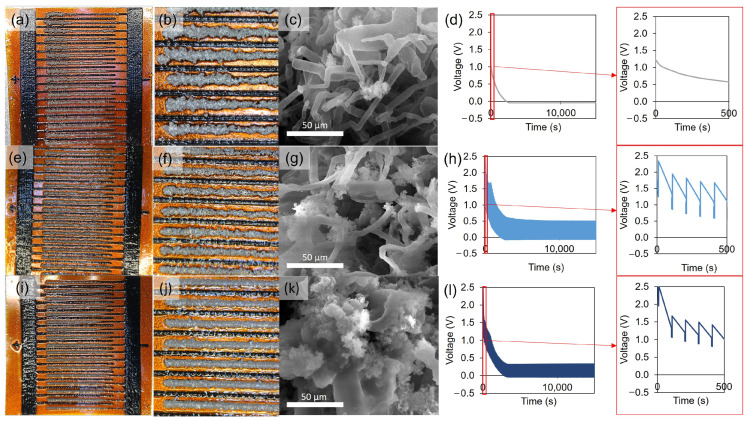
Photographs (**a**,**b**) of the electrodes and associated SEM micrograph (**c**) of electrodeposited lithium, and the plot showing electroplating overpotential (**d**) using DC plating without Ag NPs. Photographs (**e**,**f**) of the electrodes and associated SEM micrograph (**g**) of electrodeposited lithium, and the plot showing electroplating overpotential (**h**) using RPP plating without Ag NPs. Digital images (**i**,**j**) of the electrodes and SEM micrograph (**k**) of electrodeposited lithium alongside lithium electroplating overpotential plot (**l**) using RPP with Ag NPs, where all samples were plated at an (average) current density of 1 mA/cm^2^ to a capacity of 10.5 mAh/cm^2^. Scale bars in micrographs = 10 µm.

To introduce lithium metal at the patterned LIG anode, we first attempted DC plating using the setup illustrated in Figure S1. Optical (Figure 4a,b) and SEM (Figure 4c) images illustrate that the plated lithium was not uniform and porous/mossy even at a relatively low current density of 1 mA/cm^2^. More lithium is plated at the tips of the traces, and the low density (and thus larger width) of the plating results in a detrimental short-circuiting of the device. The voltage profile with plating time showing the electroplating overpotential is shown in Figure 4d.

We then explored the use of reverse pulse plating (RPP), which is a technique known to improve the plating uniformity and enhance the density of electroplated deposits [63]—including lithium [64]. This technique deposits lithium at a moderate current density (we used 1 mA/cm^2^) and then pulses a higher reverse current (10 mA/cm^2^ in our case), which preferentially dissolves any protrusions and higher surface area structures (see Table S1, Equation (S2), and associated discussion in the Supporting Information). The voltage vs. time profile (electroplating overpotential) of the RPP technique is shown in Figure 4h. As shown in Figure 4e–g, employing this technique led to significant improvements to the macroscopic uniformity as observed by optical microscopy (Figure 4e,f) and allowed us to deposit more lithium before short-circuiting the cell. However, as shown in Figure 4g, the microscopic morphology still exhibits a mossy/dendritic structure but was more compact—containing some flower-like deposits.

**Figure 5:**
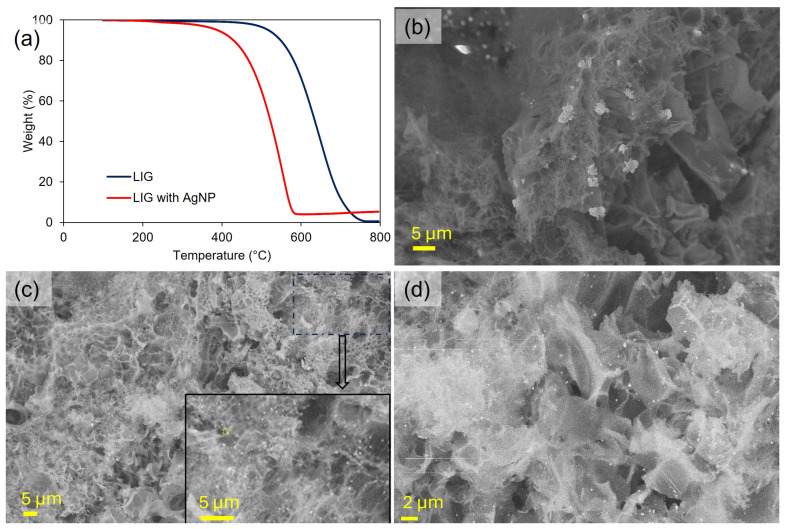
(**a**) TGA results of LIG with and without Ag NPs run in air for the determination of loading after normalization at 100 °C (~4 wt%); SEM micrograph of silver clusters (**b**) and Ag NPs (**c**,**d**) present in the LIG electrode after imbibition, shown at different magnifications.

To improve the plating morphology further, we then combined RPP with an alloying technique previously demonstrated by Cui’s group [65]. By introducing a metal that alloys with lithium during the deposition, the nucleation overpotential for plating can be reduced or effectively eliminated. Thus, in our system, Ag NPs were introduced as seeds into the porous LIG by imbibing an aqueous dispersion of Ag NPs into the porous structure and drying. TGA and Equation (S3) were used to determine that ~4 wt% Ag NPs could be introduced into the LIG by this method (Figure 5a). While the Ag NPs in dispersion were within the range of 2–4 nm in diameter as confirmed by dynamic light scattering (DLS), after depositing within the LIG, silver clusters up to 5 μm (Figure 5b,c) in size were observed due to the aggregation likely induced during the drying step and/or crystal growth from unreacted AgNO_3_ within solution. Despite some aggregation, the Ag NPs were distributed uniformly throughout the fingers as shown in Figure 5d. As shown in Figure 4i–l, this method resulted in macroscopically smooth deposits on the LIG, and dendritic structures could no longer be observed by SEM (Figure 4k). It must be noted that the addition of Ag NPs also caused a noticeable difference in the plating overpotential as shown in Figure 4l, in comparison to the RPP without Ag NPs (Figure 4h). The resulting morphology was more compact, which enables plating of up to 10.5 mAh/cm^2^ of lithium.

The voltage profiles of Li plating on LIG for DC vs. RPP and Ag NP/RPP cases are found in the Supporting Information, Figure S4a,b. DC plating without Ag NP seeding exhibited a nucleation overpotential (first plating) of ~240 mV, while the plating overpotential for subsequent cycles was ~200 mV. Plating on Ag NP-decorated LIG yields no observable nucleation overpotential, and the plating overpotential decreases significantly to ~40 mV. On the other hand, with RPP and no Ag NPs, there is still a significant nucleation overpotential of around 70 mV and a plating overpotential of about 60 mV. In the case with the RPP coupled with Ag NPs, there is no observable nucleation overpotential, and the plating overpotential is about 40 mV, similar to the DC plating with Ag NPs.

The EIS spectrum, as a function of the amount of Li plated out of the total 10.5 mAh/cm^2^, is shown in Figure S4c. The first x-intercept, corresponding to the resistance of the electrical components, including the LIG host, was initially around 94 Ω but was reduced to about 60 Ω after some Li was plated, likely due to its metallic conductivity that boosts the overall electronic conductivity of the finger. Prior to Li plating, the high-frequency semi-circle observed corresponds to the distributed resistance associated with double-layer charging in a porous network [66]. After the Li plating, this high-frequency semi-circle remains largely the same, shrinking slightly, suggesting that the Li plated does not significantly change the ionic transport within the porous LIG network. This suggests that the plated lithium does not fill all the pore space, with the majority plating on the outside of the LIG and likely only within the macropores near the surface of the fingers.

**Figure 6:**
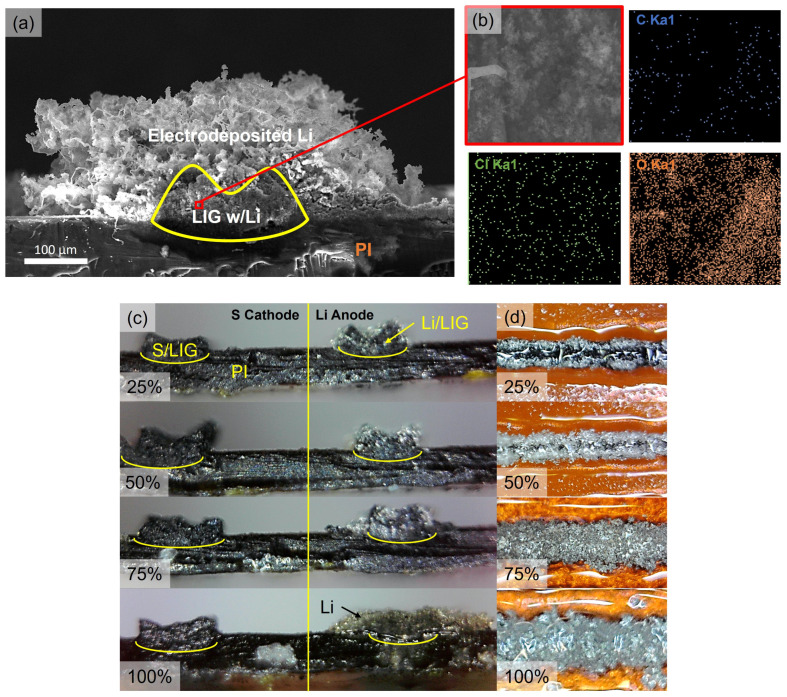
(**a**) SEM micrograph of cross-section of Li plated on the LIG anode after rinsing excess plating electrolyte and salt. Scale bar = 100 µm. (**b**) EDS elemental mapping of the interior of the LIG anode, showing that the interior LIG structure was also significant in the plating process. The atomic % of each element is as follows: C K = 13.17 at%, O K = 86.03 at%, and Cl K = 0.79 at%. This sample was plated with Ag NPs using RPP and plated to a 2.5× stoichiometric amount of Li. (**c**) Optical images of cross-sections of cathode and anode fingers: (**d**) their top-view at 25%, 50%, 75%, and 100% plating capacity of 10.5 mAh/cm^2^. The yellow curved lines are present to indicate the interface between the PI and the LIG for each electrode finger cross-section.

As shown by cross-sectional SEM imaging and EDS mapping of the plated Li in Figure 6, the lithium can only be observed to plate around the LIG, and it is difficult to conclude whether the Li plates within the porous LIG itself (the EIS results would suggest it does not). EDS carried out in the LIG cross-section indicates the presence of chlorine (0.8 at%) and, to a greater extent, oxygen (86 at%), which suggests the existence of decomposition products corresponding to the plating salt (LiClO_4_) and carbonate-based solvent within the pores [67]. While this could be due to the SEI formed on Li metal, it may also correspond to the SEI formed on the LIG itself during the lithium intercalation that precedes plating, suggesting the significance of the interior structure in the plating process as well. It is possible that chloride may simply be adsorbed to the high-surface-area carbon. Figure 6c,d shows optical microscope cross-sections and top views of the plated anodes at different levels of plating. The LIG traces are raised at the edges compared to the center where the laser power is highest and likely causes some ablation. At these edges, which are closer to the bulk lithium piece during plating, Li plating begins and covers the entire trace at around 75% of the plating capacity.

### 3.4. LIG-Based Full Cell

**Figure 7:**
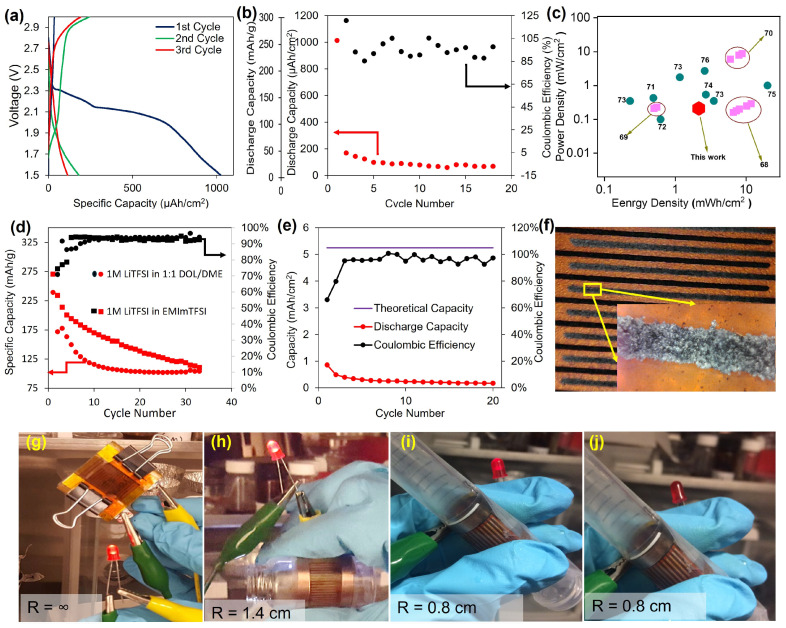
(**a**) Typical voltage curves of the first three discharge and charge cycles. (**b**) Galvanostatic cycling results of a full, LIG-based cell. Red arrow indicates capacity data. Black arrow indicates Coulombic efficiency data. (**c**) Areal Ragone plot comparing LSFB (red diamond) to thin-film/flexible microbatteries present on the market (squares) [69,70,71] and in the literature (circles) [72,73,74,75,76,77]. (**d**) Cycling results for S/LIG material vs. bulk Li anode in a coin cell configuration in two different solvents, 1 M LiTFSI in 1:1 DOL/DME (ether-based electrolyte, circle markers) and 1 M LiTFSI in EMImTFSI (RTIL-based electrolyte, square markers). Red arrow indicates capacity data. Black arrow indicates Coulombic efficiency data. (**e**) Cycling results of Li vs. Li on LIG fingers, both plated to a capacity of 5.25 mAh/cm^2^, which is displayed as the purple horizontal line that also indicates what the theoretical, expected capacity is for each cycle. (**f**) Digital image of Li anode fingers after completely stripping Li metal to 1 V vs. Li, indicating the gray SEI remaining. Digital images of a bending test with the LIG-based LSFB, where a red LED is connected to the device and the intensity of the light is an indicator of the current passing to the LED. The bending radii tested were (**g**) ∞ (flat configuration), (**h**) 1.4 cm (around a 20 mL scintillation vial), and (**i**) 0.8 cm (around a 15 mL conical bottom centrifuge tube). The LED disconnected in (**j**) to show that the LED in (**i**) is on, despite it being dimmer than in (**g**) or (**h**).

Figure 7a indicates the first three charge and discharge curves of the full interdigitated Li-S cell using the high-sulfur-loading cathode (75 wt% sulfur, 3.9 mg/cm^2^) and the optimized anode (Ag NP seeding combined with RPP) plated to 10.5 mAh/cm^2^ (approximately 2× stoichiometric lithium relative to the sulfur cathode). The first discharge yields a specific capacity of over 1000 µAh/cm^2^, or equivalent to ~250 mAh/g. The voltage profile is very typical of Li-S chemistry systems, with an upper plateau of around 2.3 V and a lower plateau around 2.1 V, corresponding to the solid–liquid transition of Li_2_S_8_ and the reduction of the long-chain polysulfide species (*n* = 6 to *n* = 8) to the shorter-chain polysulfide species (*n* = 4) for Li_2_S_n_, respectively [68]. However, upon the first charge and thereafter, the capacity of the battery suffers from a very rapid drop, with a capacity retention around 17% at the second cycle (Figure 7a,b).

The initial capacity that is obtained with the LIG-based full cell exhibits an energy density and power density that are comparable to other microbatteries and thin film or flexible batteries that have been commercialized or presented in the literature (Figure 7c). This is remarkable, especially considering the ease of solution-based processing compared to that of the other complex and high-capital methods for typical cells. The considerable energy density achieved is due to the inherent benefit of the high capacity of the lithium-sulfur chemistry, but the power density is limited by the large distance between the electrode fingers in addition to the viscous RTIL-based electrolyte used, which both contributes to large ohmic drops during cycling.

### 3.5. Half-Cell and Symmetric Cell Failure Analysis

To investigate the capacity drop, we aimed to deconvolute the potential sources of parasitic reactions in the LSFB. Since our system, due to the low amount of excess Li, is effectively a full cell, the rapid capacity loss could be due to either the anode, the cathode, or both.

To study the cathode in more detail, a coin cell was prepared using the LIG/S composite and tested in a half-cell against lithium foil (several orders of magnitude excess as is typical). As shown in Figure 7d, the specific capacities obtained in both ionic liquid and a traditional ether-based electrolyte are 270 and 239 mAh/g, respectively. This is significantly lower than the theoretical capacity of 1672 mAh/g. We attribute this to the relatively low surface area (180 m^2^/g) of the LIG compared to the carbonaceous materials typically used in Li-S batteries such as activated carbon [78], carbon nanotube [79], and non-agglomerated graphene-based electrodes, which can achieve >1000 m^2^/g [61,80] (see Figure S6 for voltage–capacity profiles and associated discussion). For this material, an optimized performance might be achieved at lower loading and sulfur content, but this requires further development. The obtained capacity in both electrolytes is initially very similar, while the ether-based electrolyte cell suffers a more rapid capacity fade, likely due to the increased solubility of sulfur and polysulfides in comparison to the RTIL [81,82,83]. Each electrolyte reaches a similar capacity of ~105–110 mAh/g after 33 cycles. Such a capacity fade is expected since we did not incorporate any specific polysulfide scavengers in the battery. Comparing the capacity fade between the first and second cycle of these half-cells (~86% and ~72% for the RTIL-based and ether-based electrolytes, respectively) and the interdigitated full cell (~17%) suggests that the severe cycle-life problem may originate from the anode in the interdigitated system.

Typically, Li-S batteries undergo several initial cycles where the cell’s capacity slightly decreases due to the formation of a stable SEI, which occurs at the expense of electrolyte decomposition and irreversible reactions with the Li metal. In this instance, since the Li metal is introduced through electrodeposition, an inherent SEI layer should be present. To verify this, we completely stripped the electrodeposited lithium from LIG and observed a visible gray film over the LIG electrodes, which we attribute to the SEI (Figure 7f). Despite this inherently formed SEI, it was either insufficient in thickness or unstable, leading to a poor performance in protecting the metallic Li plated.

To determine how much Li could be cycled after the electroplating process and to avoid complications that could stem from the cathode, we plated Li metal on both sets of fingers with equivalent capacity (anode and cathode) to carry out symmetric Li-Li testing. As shown in Figure 7e, the first discharge capacity was 0.31 mAh, while the capacity originally plated was 1.874 mAh. This is 16.5% of the theoretical plating capacity. After a slower, gradual decrease in capacity over the next five cycles, the capacity remains relatively constant, suggesting that a significant amount of sacrificial Li was necessary to produce a stable SEI after changing from the plating (carbonate-based) to the cycling electrolyte (RTIL-based). The voltage vs. plating and stripping capacities for both cases are shown in Figure S6.

In the full cell, since ~3.75 mAh of lithium anode was initially plated, if only 16.5% of this lithium is accessible, there would still be an anode capacity of 0.618 mAh. This is much lower than the theoretical sulfur capacity of 3.91 mAh initially patterned on the cathode fingers. Since we were only able to plate 1.87 mAh of lithium on each finger for the Li-Li symmetric cell measurements, half that of the symmetric cell to avoid cell shorting, it is possible that the usable lithium after plating is even less than 16% when further lithium is deposited. On the other hand, since the sulfur utilization at the cathode was less than the theoretical, and since ~270/1672 × 6.52 = 1.05 mAh/cm^2^ (or 0.63 mAh) of sulfur cathode is useable capacity upon the initial cycle and matches well with the measured full cell capacity on the first cycle, this means that considering the usable capacity of the anode and cathode, the cell is nearly balanced at least upon the first cycle. In subsequent cycles, the large drop in the full cell capacity is likely due to a combination of both cathode and anode degradation modes with both degradation modes being accelerated compared to the half-cell configuration performance. This likely happens due to the relatively large surface area of the lithium deposited, which can more rapidly consume polysulfides and become passivated compared to the half-cell case where a flat lithium foil was used.

### 3.6. LIG-Based Cell Flexibility

To verify the flexibility of the device, the LSFB was connected to a red LED in three different configurations with three different radii of curvature (Figure 7g–i). The LED was very brightly lit in the case where the device was flat in the cycling setup described in Figure S5 and with a radius of curvature of 1.4 cm. While the LED’s brightness was much lower in the case of the radius of around 0.8 cm, this is mainly due to the difficulty in retaining the liquid electrolyte around the LIG fingers, resulting in a diminished current and accessible active material. It is then clear that the device is capable of operation within a bent configuration, though currently, it is limited by the electrolyte. This is expected to be a non-issue in the case with a gel polymer electrolyte or ionogel-based electrolyte, which would enable a fully conformable electrolyte that also maintains the ionic contact within the device. In addition to the benefit of constant ionic contact regardless of the bending radius, the use of a gel polymer electrolyte with suitable functionalities in the polymer matrix and additives should also be able to prevent the polysulfide shuttling and hinder the growth of lithium dendrites and minimize safety risks. In future work, electrolyte additives that stabilize the SEI should also be investigated. Often, lithium nitrate (LiNO_3_) is used in ether-based electrolytes, which helps to passivate the lithium anode and slows/prevents polysulfide shuttling [84]. We did not test this in the present study because we found limited solubility of LiNO_3_ in the IL electrolyte. Furthermore, the EMImTFSI electrolyte may not be the best choice of electrolyte for this system. In one report, the EMIm^+^ cation was found to reduce and form an unstable SEI layer on graphite [85]. While this report was not focused on the effects of using RTIL for Li plating, the finding of the cation instability could suggest that EMIm^+^-based RTILs may not be suitable for Li plating in our flexible Li-S system.

## 4. Conclusions

The use of LIG electrodes in fabricating Li-S batteries has been demonstrated as feasible through a novel two-step heterogeneous nucleation/growth and imbibition technique, combined with lithium electrodeposition aided by RPP and Ag NP seeding. This approach enables denser films on porous LIG fingers and achieves capacities comparable to or exceeding those of flexible thin-film batteries produced using cleanroom techniques, as shown in the Ragone plot (Figure 7c). However, the performance is constrained by the LIG pore structure’s impact on the molten sulfur distribution during melt imbibition and the efficiency of Li metal plating on the LIG traces. Enhancing the LIG pore structure and the sulfur deposition process is essential.

The poor cycling performance of the RPP/Ag NP-aided electrodeposited Li anode indicates that challenges and failure mechanisms would persist even with sputtered Li. This highlights the need for further research on passivating the Li metal anode against the electrolyte, especially in Li-limited Li-S batteries.

The presented methods offer a promising proof of concept for flexible energy storage devices and operando spectroscopic studies enabled by the open architecture. Such studies are critical for investigating LiPS migration and other challenges to better understand and address the operational and failure mechanisms in near-stoichiometric lithium cells. These insights are essential for advancing practical Li-S batteries with energy densities closer to their theoretical potential.

## Data Availability

Data are available upon reasonable request from the authors.

## Supplementary Materials

The following supporting information can be downloaded at: https://www.mdpi.com/article/10.3390/nano15010035/s1, Figure S1: Diagram of the side view of lithium plating setup; Figure S3: Plating voltage curves of lithium onto the LIG; Figure S2: SEM images of cross-sections of several adjacent anode–cathode pairs illustrating their widths; Figure S3: Ethanol vapor adsorption analysis; Figure S4: Plating voltage curves of lithium onto the LIG; Figure S5: Cycling setup of flexible Li-S batteries; Figure S6: Analysis of charge–discharge profiles for LIG/S half-cell vs. bulk lithium; Figure S7: Examples of lithium plating–stripping curves in different configurations; Table S1: Lithium plating parameters chosen for the RPP technique; Table S2: Details and parameters for the cited devices in Figure 7c; Equations and additional discussion. Refs. [86,87,88,89,90] are cited in Supplementary Materials.
