# Supplementary material for: Patterning Planar, Flexible Li-S Battery Full Cells on Laser-Induced Graphene Traces
Source: Nanomaterials (Basel). 2024 Dec 29;15(1):35. doi: 10.3390/nano15010035 (PMC11723327; doi:10.3390/nano15010035)
Supplement: Supplementary file 1 [file nanomaterials-15-00035-s001.zip › nanomaterials-3398995-supplementary.pdf]

Supporting Information for

Patterning Planar, Flexible Li-S Battery Full Cells on Laser-Induced Graphene Traces

Irene Lau [1], Adam I. O. Campbell [1], Debasis Ghosh [2] and Michael A. Pope [1,*]

[1] Quantum Nano Centre, Department of Chemical Engineering, University of Waterloo, Waterloo, ON N2L 3G1, Canada
[2] Centre for Nano & Material Sciences, Jain (Deemed to be University), Jain Global Campus, Bangalore 562112, India; g.debasis@jainuniversity.ac.in or debasisghosh88@gmail.com
* Correspondence: michael.pope@uwaterloo.ca; Tel.: +1-519-888-4567 (ext. 30153)

TGA Equation for the Determination of the Weight Fraction of S in the LIG:

$$\text{wt\% of S} = \frac{\text{wt\% remaining at 100°C} - \text{wt\% remaining at 800°C}}{\text{wt\% remaining at 100°C}} - \text{wt\% loss of LIG in } N_2 \quad \text{(S1)}$$

Anode: Li Electrodeposition onto LIG

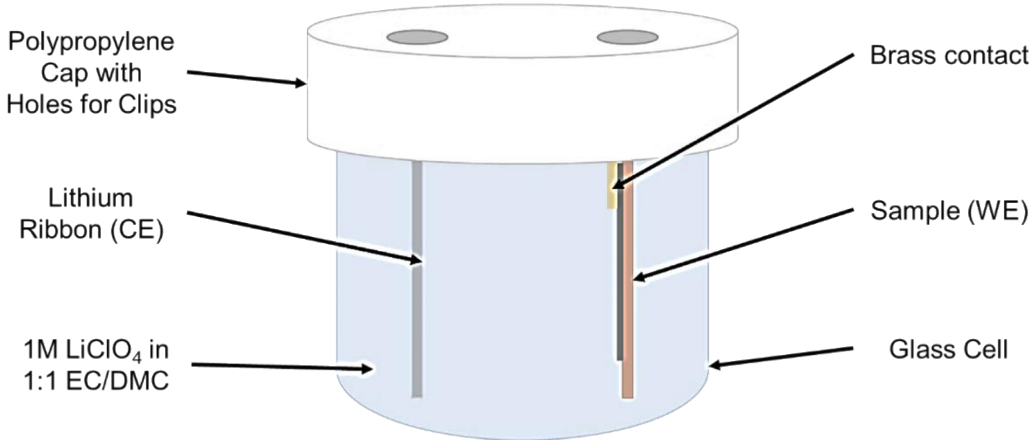

Figure S1. Diagram for the side view of lithium plating set up, where the distance between the counter electrode (CE) and working electrode (WE) is 3 cm. A piece of lithium ribbon (length x width = 5 x 4 cm) was used as the counter electrode and the working electrode is the anode of the sample itself. Both CE and WE were contacted with a piece of brass while set 3 cm apart in a glass holder in a bath of the plating electrolyte 1M LiClO4 in 1:1 EC/DMC.

Both direct current (DC) and a reverse pulse plating (RPP) techniques were applied to electrodeposit lithium onto the LIG. For using RPP, according to Chandrasekar et al.[1], the specific cathodic and anodic currents and times must be chosen with the consideration of the duty cycle as well as the amount of time required to charge the electrical double layer on each of the pulses. For the RPP technique, the average current density, I_A, is defined as:

$$I_A = \frac{I_C T_C - I_{AA} T_{AA}}{T_{AA} + T_C} \quad , \qquad \text{(S2)}$$

where I_C is the forward, cathodic current and T_C is the length of the cathodic current pulse in time, I_{AA} is the reverse, anodic current and T_{AA} is the length of the anodic current pulse in time. Table S1 indicates the specific parameters used for lithium plating with the RPP technique, based on a study that was conducted by Yang et al.[2] in 2014 to determine the effects of various parameters on the morphology of plated lithium as well as the cycling efficiency of Li/Li symmetric cells. The values as listed in Table S1 yielded the most compact morphology while also giving the highest cycling efficiency, indicative of the lowest rate of loss of lithium due to reactions with the electrolyte or dendrites that form and break off.

Table S1. Lithium plating parameters chosen for the RPP technique based on[2].

Parameter	Value
Cathodic current, I_C (mA/cm^2)	1
Cathodic current pulse length, T_C (ms)	5000
Anodic current, I_{AA} (mA/cm^2)	10
Anodic current pulse length, T_{AA} (ms)	20

Examples of cathode and anode widths:

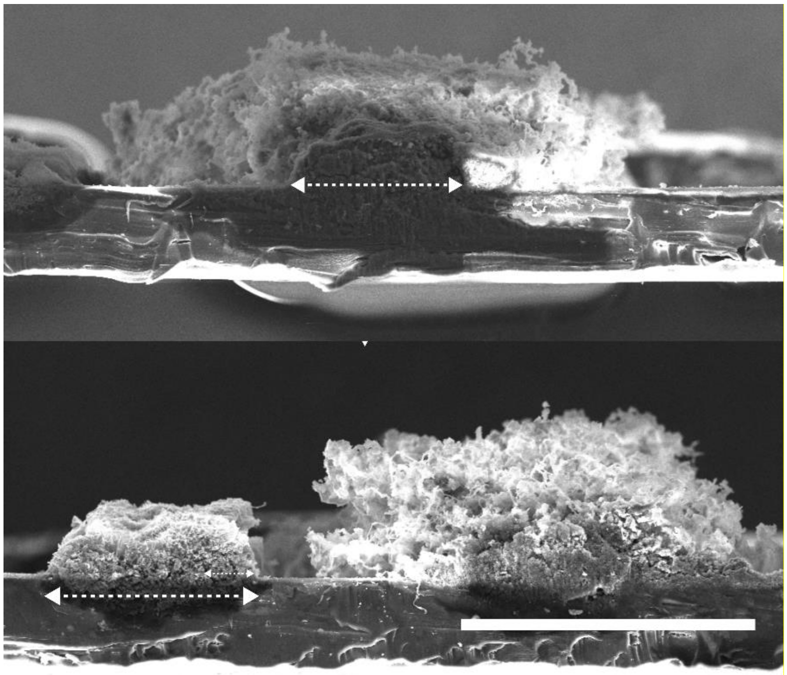

Figure S2: SEM images of cross-sections of several adjacent anode-cathode pairs illustrating their widths. Scalebar is 400 µm. Cathode is on bottom left and measures 292 µm. Anode width is shown more clearly on top right and measures 210 µm in width (not considering plated lithium).

Ethanol adsorption analysis

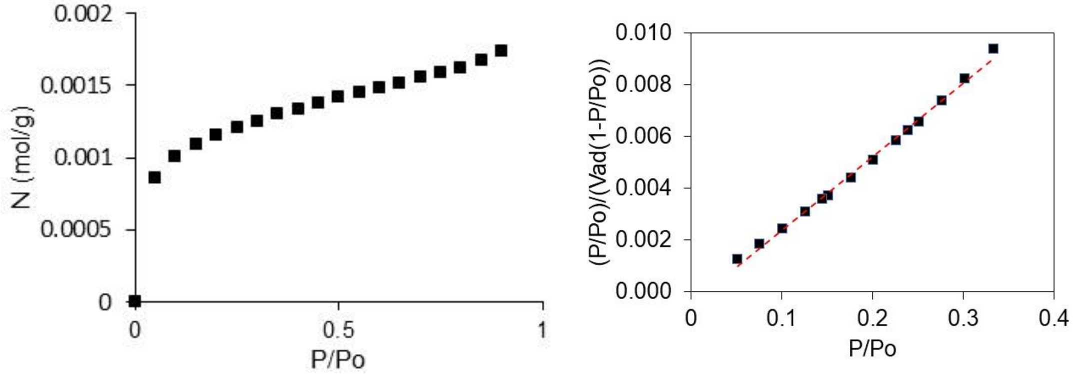

Figure S3: Ethanol vapor adsorption data (left) with corresponding BET transform (right). The fit to the BET transform is given below and was used to estimate the SSA.

$$\frac{P/P_o}{V_{ad}\left(1-P/P_o\right)} = \frac{C-1}{CV_{ad,mon}}\left(P/P_o\right) + \frac{1}{CV_{ad,mon}} \rightarrow y = 2.84635 \cdot 10^{-2}x - 4.74421 \cdot 10^{-4}$$

TGA Equation for the Determination of the Weight Fraction of Ag NP in the LIG:

$$\text{wt\% of Ag NP} = \frac{\text{wt\% remaining at 800°C}}{\text{wt\% remaining at 100°C}} - \text{wt\% loss of LIG in air} \qquad \text{(S3)}$$

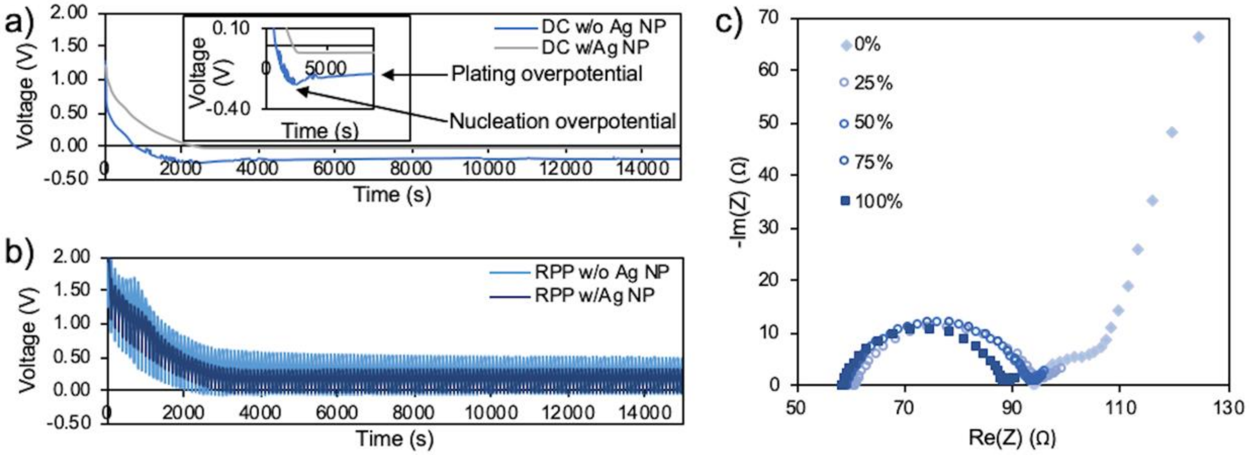

Figure S4. Plating voltage curves of lithium onto the LIG using a) DC plating (inset: zoom in to the initial stages of plating to highlight difference between plating and nucleation overpotentials) and b) RPP, with and without Ag NP as indicated; c) EIS spectra as a function of the total 10.5 mAh/cm^2 of lithium plated using RPP with Ag NP and an average current density of 0.1 mA/cm^2.

Electrochemical Testing:

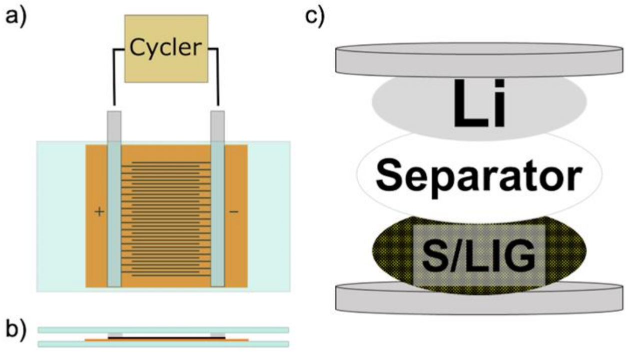

Figure S5. Cycling setup of the flexible Li-S batteries with where a) is the top view and b) is the side view. The device is contacted by copper contacts and held together by clips to contain the cycling electrolyte. Batteries were cycled between 1.5-3.0 V at 0.1 C in 1M LiTFSI in EMImTFSI; c) Cell configuration for S/LIG vs Li coin cells where the

electrolytes used were 1M LiTFSI in EMImTFSI and 1M LiTFSI in 1:1 DOL/DME. Batteries were also cycled between 1.5-3.0 V at 0.1 C.

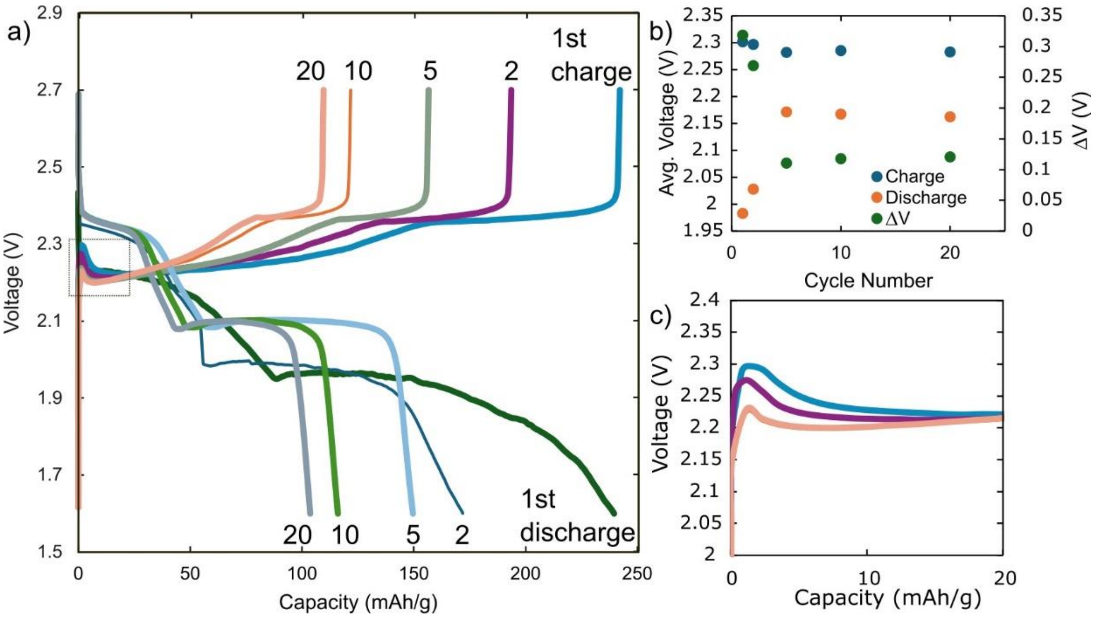

Figure S6: Analysis of charge-discharge profiles for S/LIG half-cell vs. bulk lithium anode: a) Examples of charge-discharge profiles at various cycle numbers at a current of 0.5 mA; b) Magnified view of initial polarization upon charging; c) Average charge and discharge voltage and the difference between the average charge and discharge voltages (ΔV) as a function of cycle number.

Figure S5 shows the voltage-capacity profiles for the S/LIG vs. bulk Li metal corresponding to the cycling data presented in the main text Figure 7. The discharge plateaus for the first and second cycle are significantly lower (by >150 mV) compared to subsequent cycles. This indicates an initial activation of the S/LIG cathode possibly through a redistribution of sulfur deep in micropores that may be initially blocked and leading to higher IR drop in the electrode network. As shown in Figure S5a,b from the 3rd cycle on, there is no significant change in the discharge voltage and the average voltage gap between charge and discharge (ΔV). Only a consistent and significant capacity fade is observed through all cycle which is likely due to the rapid dissolution in the relatively open porosity of the LIG. As mentioned in the main text, we did not implement any strategies to confine polysulfides in the cathode and these diffuse into the bulk of the electrolyte and eventually react at the anode. In many of our full cells, we observed colour change in the electrolyte. In fact, we hope that this interdigitated platform could be an interesting system

for future work on operando spectroscopic analysis of speciation. As illustrated in the magnified volage-capacity profile shown in Figure S6c, we also observe polarization upon switching from discharge to charge cycles which decrease in magnitude with cycle number. This is often associated with the sluggish conversion of Li_2S. The capacity fade combined with the observed reduction in polarization might indicate that Li2S becomes irreversibly deposited on the electrode network. Thus, we are likely losing sulfur to shuttling as well as irreversible Li_2S conversion.

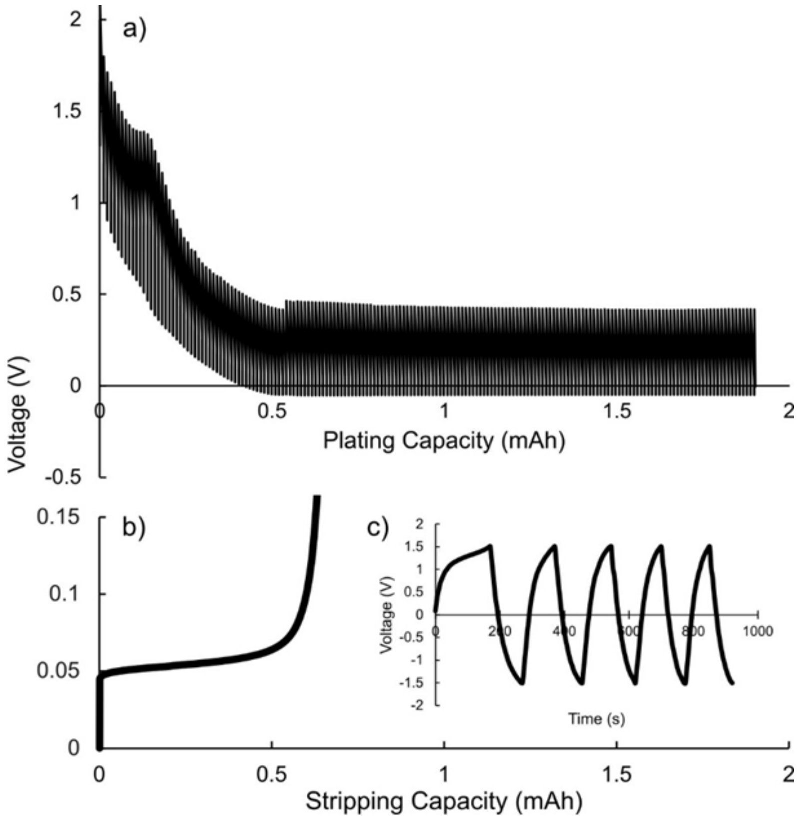

Figure S7. Examples of lithium plating-stripping curves in different configurations. a) Initial RPP curve to a total of 1.9 mAh on the LIG fingers. About 0.4-0.5 mAh of this capacity is used to lithiate the carbon and form SEI while 1.4-1.5 mAh is used to plate lithium and form SEI. b) The same electrode in a) immediately stripped in the same plating electrolyte (1:1 EC/DMC in 1 M LiClO4). A stripping overpotential of ~50 mV is observed and only ~0.5 mAh of lithium can be stripped off the LIG in this plating electrolyte. c) Charge-discharge curves of symmetric lithium cell comprised of LIG plated on both anode and cathode fingers (1.874 mAh of lithium each) in cycling electrolyte (1M LiTFSI in EMImTFSI). There is a significant overpotential of ~1 V and a stripping/plating plateau is only observed on the first cycle. Afterwards, the capacity rapidly fades, and the electrodes exhibit only capacitor-like charge/discharge profiles.

Table S2. Details and parameters for the cited devices in **Figure 7c**

#	Ref.	Name of Device	Dimensions (*l* * *w* * *t*, in cm)	Type of Chemistry	Method of Fabrication	Nominal Voltage (V)	Energy Density (mWh/cm^2)	Energy Density (mWh/cm^3)	Power Density (mW/cm^2)	Power Density (mW/cm^3)
1)	[3]	Bright Volt	2.2 * 2.9 * 0.045	Li-ion, solid state	Unknown	>3	6.58	146.29	0.16	3.66
			2.2 * 2.9 * 0.045				7.99	177.64	0.20	4.44
			2.2 * 2.9 * 0.054				11.76	217.69	0.29	5.44
			4.5 * 2.3 * 0.045				7.25	161.03	0.18	4.03

			4.5 * 2.3 * 0.045				10.14	225.44	0.25	5.64
2)	4	Panasonic CG	2.85 * 3.9 * 0.055	Li-ion	Unknown	3.8	5.98	108.78	5.98	108.78
			3.5 *5.5 * 0.055				7.90	143.57	7.90	143.57
			4.0 * 6.5 * 0.055				8.77	135.00	8.77	135.00
3)	5	Foldable LiS Battery	? * ? * 0.004	LiS	Slurry casting	2.1	2.70	675.00	0.54	135.00
4)	6	Microbatteries from [6]	? * ? * 0.0015	Li-ion	Micro-fabrication	>3	0.23	150.00	0.35	230.00
			? * ? * 0.00126	Li-ion	Micro-fabrication	>3	0.01	6.00	93.24	74000.00
			? * ? * 0.0065	Li-ion	Micro-fabrication	>3	0.02	3.30	0.18	27.70

			? * ? * 0.05	Li-ion	Micro-fabrication	>3	3.50	70.00	0.35	7.00
			? * ? * 0.05	Li-ion	Micro-fabrication	>3	1.16	23.10	1.75	35.00
			? * ? * 0.018	Li-ion	Micro-fabrication	>3	0.03	1.70	0.01	0.40
			? * ? * 0.020	Ni-Zn	Micro-fabrication	1.65	0.0002	0.10	0.03	1.70
5)	7	Free-Form Flexible Battery	0.225 * 0.17 * 0.003	Li-ion	Micro-fabrication	3.3	0.49	16.17	0.43	143.10
6)	8	EnerChip CC	0.225 * 0.17 * 0.01	Li-ion	Unknown	3.8	0.50	4.97	0.21	20.55
			0.57 * 0.61 * 0.02			3.8	0.55	27.32	0.23	11.31
7)	9	Thin Film Li	1 * 1 * 0.00025	Li, Li-ion	Micro-fabrication	3.8	0.62	2477.60	0.10	412.93

		and Li-ion								
8	10	Flexible solid state Li-ion micro battery		Li-ion	Mask-assisted fabrication	1.7		146.3		8800 (8.8 W cm^{-3})
9	11	Li-ion Microbatteries	0.6*0.2*0.07	Li-ion	Imprint Lithography	>2	34.44 (1.24 J cm^{-2})			75.5
10	12	Flexible Li-ion batteries			Electrostatic spray deposition	>3.8	305.49 Wh/kg			
11	13	Li-ion Microbatteries		Li-ion	Holographic patterning	>2	0.0065 (6.5 μWh cm^{-2})		3.6 (3,600 μW cm^{-2})	
12	14	3D printed	total	Li-ion	Direct ink writing	1.8	20		1	

		Li-ion Batteries	volume < 1 mm^3							
13	[15]	3D Li-ion Microbatteries		Li-ion	3D printing technique	>1.8	2.6		2.7	
14	[6]	3D Li-ion Microbatteries		Li-ion	Electrodeposition	>2	0.0006 (0.6 μWh cm^{-2})		7.4	
15	[16]	Li-ion Microbatteries		Li-ion	Electrodeposition	2.5	0.0084 (8.4 μWh cm^{-2})		7.4	
16	This work	LSFB	1.2 * 16*(0.0287) * 0.0073	LiS	Laser scribing, solution-based processing	2.1	2.15	534.42	0.21	53.44

Determination of Dimensions for Energy and Power Density in Table S2

1. **Bright Volt:**

   No specific internal dimensions are given in the product description and thus the external dimensions given in the product specification are used in the calculations.

2. **Panasonic CG:**

   No specific internal dimensions are given in the product description and thus the external dimensions given in the product specification are used in the calculations.

3. **Foldable Li-S Battery:**

   The only dimension given was the thickness of the cathode slurry layer with the CNT, 40 μm. This value was used in combination with the areal energy and power densities in order to determine the volumetric energy and power densities.

4. **Microbatteries from Pikul:**

   The microbatteries from Pikul et al.'s paper and also some referenced Li-ion-based microbatteries are also used in this work for comparison. Table 1 of Pikul's paper includes the thicknesses of the electrodes and the respective energy densities and C-rates for the selected batteries for comparison. The values included in the table were also used in this work for the calculation of the volumetric and areal energy and power densities.

5. **Free-Form Flexible Battery:**

   The exact dimensions given in the work included the full dimensions of the sandwich cell but layer-specific dimensions were not given and thus the full device dimensions were used in the calculation of the energy and power densities.

6. **EnerChip CC:**

No specific internal dimensions are given in the product description and thus the external dimensions given in the product specification are used in the calculations.

7. **Thin Film Li and Li-ion Battery:**

   The area of 1 cm^2 and a thickness of 2.5 μm was given for the cathode. Thus, these were the dimensions used in the calculation of the energy and power densities.

8. **LSFB:**

   The overlapping (1.2 cm overlap) area between the two sets of electrode fingers were utilized in the calculations only. Also, as the cell has not been optimized in its form with respect to the electrode pitch, the total overlapping cathode finger area and volume was used, where there are 16 fingers, each with a length of 1.2 cm overlap and a width of 287 μm and a thickness of 73 μm.

1. **References**

1. Chandrasekar, M. S.; Pushpavanam, M., Pulse and pulse reverse plating—Conceptual, advantages and applications. *Electrochimica Acta* **2008,** *53* (8), 3313-3322.
2. Yang, H.; Fey, E. O.; Trimm, B. D.; Dimitrov, N.; Whittingham, M. S., Effects of Pulse Plating on lithium electrodeposition, morphology and cycling efficiency. *Journal of Power Sources* **2014,** *272*, 900-908.
3. "Product Matrix | BrightVolt." [Online]. Available: https://www.brightvolt.com/productmatrix/. [Accessed: 06-Jul-2018].
4. "Panasonic Develops Bendable, Twistable, Flexible Lithium-ion Battery | Headquarters News | Panasonic Newsroom Global." [Online]. Available: https://news.panasonic.com/global/press/data/2016/09/en160929-8/en160929-8.html. [Accessed: 06-Jul-2018].
5. Li, L.; Wu, Z. P.; Sun, H.; Chen, D.; Gao, J.; Suresh, S.; Chow, P.; Singh, C. V.; Koratkar, N., A Foldable Lithium–Sulfur Battery. *ACS Nano* **2015,** *9* (11), 11342-11350.
6. Pikul, J. H.; Gang Zhang, H.; Cho, J.; Braun, P. V.; King, W. P., High-power lithium ion microbatteries from interdigitated three-dimensional bicontinuous nanoporous electrodes. *Nature Communications* **2013,** *4* (1).
7. Kutbee, A. T.; Ghoneim, M. T.; Ahmad, S. M.; Hussain, M. M., Free-Form Flexible Lithium-Ion Microbattery. *IEEE Transactions on Nanotechnology* **2016,** *15* (3), 402-408.
8. "Thin Flexible Batteries | Battery Solutions." [Online]. Available: https://www.bluesparktechnologies.com/index.php/products-and-services/batteryproducts. [Accessed: 06-Jul-2018].
9. Bates, J., Thin-film lithium and lithium-ion batteries. *Solid State Ionics* **2000,** *135* (1-4), 33-45.
10. Zheng, S.; Wu, Z.-S.; Zhou, F.; Wang, X.; Ma, J.; Liu, C.; He, Y.-B.; Bao, X., All-solid-state planar integrated lithium ion micro-batteries with extraordinary flexibility and high-temperature performance. *Nano Energy* **2018,** *51*, 613-620.
11. Sun, P.; Li, X.; Shao, J.; Braun, P. V., High-Performance Packaged 3D Lithium-Ion Microbatteries Fabricated Using Imprint Lithography. *Advanced Materials* **2020,** *33* (1).
12. Lee, S. C.; Jeong, J.; Park, H. G.; Min, B.-C.; Chan Jun, S.; Chung, K. Y., Binder-assisted electrostatic spray deposition of LiCoO2 and graphite films on coplanar interdigitated electrodes for flexible/wearable lithium-ion batteries. *Journal of Power Sources* **2020,** *472*.
13. Ning, H.; Pikul, J. H.; Zhang, R.; Li, X.; Xu, S.; Wang, J.; Rogers, J. A.; King, W. P.; Braun, P. V., Holographic patterning of high-performance on-chip 3D lithium-ion microbatteries. *Proceedings of the National Academy of Sciences* **2015,** *112* (21), 6573-6578.
14. Wei, T. S.; Ahn, B. Y.; Grotto, J.; Lewis, J. A., 3D Printing of Customized Li-Ion Batteries with Thick Electrodes. *Advanced Materials* **2018,** *30* (16).
15. Sun, K.; Wei, T. S.; Ahn, B. Y.; Seo, J. Y.; Dillon, S. J.; Lewis, J. A., 3D Printing of Interdigitated Li-Ion Microbattery Architectures. *Advanced Materials* **2013,** *25* (33), 4539-4543.
16. Pikul, J.; Zhang, H. G.; Cho, J.; Braun, P.; King, W., High power lithium ion microbatteries with lithographically defined 3-D porous electrodes. In *2013 IEEE 26th International Conference on Micro Electro Mechanical Systems (MEMS)*, 2013; pp 857-860.
